# East and West

**Published:** 1887-12-17

**Authors:** Leslie Keith

**Affiliations:** Author of "The Chilcotes," "Uncle Bob's Niece," etc.


### EAST AND WEST.

BY LESLIE KEITH.

Author of "The Chilcotes," "Uncle Bob's Niece," etc.

(*Continued from p.* 186.)

### CHAPTER XII.—DAVID'S HOUR OF TRIUMPH.

ALICIA piloted the pair herself down to the cottage by the river, and arriving there announced that she intended to remain. It was found, indeed, that a bed-room was prepared for her, and that a young woman, drafted from the household in Park Lane, had been told off to wait upon the exiles.

So the Frankland mansion was at last abandoned—brown paper shrouded the upper windows, and the servants held unchecked revel below, now that their young mistress had carried her eccentricities out of town. Alicia had reconciled this step very easily to her conscience. She had promised her father that she would not sleep in Bethnal Green, but Rose Cottage was not Bethnal Green. Besides, she had taken pains, after establishing herself and her guests, to obtain his consent. So life took a new departure for the three in a world widely different from that they had left—here the air blew sweet and clean, the river sang to them, a little lawn sloping to the water was made gay for them with bright beds of flowers. It was a modest home, but it was very peaceful, and even in the coolness of the early September morning you could hear the birds singing almost as if they had forgotten that it was not June.

A perfect place for a poet, surely—a place to inspire him, and make him dexterous in new rhymes. Yet, for the first week David Jardine was restless and unhappy, and on the whole "Gey ill to live wi'," like his bigger countryman. By the second week, however, those proof-sheets which are the glory of the youthful author and the torment of his more experienced brother, began to arrive, and he was content. To see his thoughts in print for all the world to read was an exquisite joy; he did not remember the moral of the sheets in which Mrs. Carter wrapped her butter; he only saw fame writ large on his horizon; fame and fortune, and all the sweets of life that were never to be his.

Mary sat all day long on the lawn by his couch with her sewing, ready to anticipate his smallest wish—ready to listen all day to his verses and to think them beautiful—as beautiful as the poet himself with the refining touch of sickness on his features. Love does not blind, but it makes allowance easy; he was vain, shallow, selfish, and not even very clever—at best but a pale echo of other and greater singers, but he was something more and better as well, and it was the something more and better on which she dwelt as only one's feminine kin find it possible to do.

Alicia was patient with the invalid too, patient as one is with a fretful child. She was very sorry for him. Sorry that he was going away from a world that was beautiful in spite of all the wrong and suffering in it, but for the most part she left him with Mary.

She did not often join the pair on the lawn, and she made no offer to help the sewing that Mary still insisted on continuing.

She too, was restless, and she had not David's supreme belief in herself to sustain her. For her too, the arrival of the post made an epoch in her day. There were letters from abroad to account for this—letters from the travellers who had extended their wanderings beyond Homburg. From their own account of themselves they were having a very good time. Jack and Kitty had diligently conjugated the verb *aimer* under the moon of Switzerland, and on the lakes of Italy; and Mr. Frankland had smoked many thoughtful cigars over Alicia's hot and earnest messages from out the East. But Alicia did not envy them their liberty to stray; her own explorations were infinitely more interesting to her, and she hotly repudiated the idea of joining her father and sister when they suggested this course. If the truth were known, she was pining to be back on the scene of her summer adventures.

This avowal is made with diffidence, because it implies a fatal want of taste in a young lady who has been brought up in Park Lane, and has never known any but the best people. It is however, strictly true, and it proves Mary Jardine to have possessed a certain shrewdness when she warned her cousin:

"A day may come when you will not find it possible to forsake us."

There were other letters for Miss Frankland besides those with a foreign postmark—letters written in that room where Felix Mervyn had been wont to burn the midnight oil of a student. They were strictly business letters, and the affairs must surely have been rather pressing that required the aid of pen and ink as well as of personal interviews, for the busy doctor made a habit of visiting his patient two or three times a week in the cottage by the river. He could do nothing for David now, and nothing for Mary, for he could not give her hope, and she knew it; therefore, he probably had another motive in going so often.

The truth was, Alicia had handed over all her embryo schemes to the doctor when she exiled herself from Bethnal Green. Among these plans was that rescuing of country people, and restoring of them to their own homes, that had seemed to her a needful thing to be done. She had begun with the young couple who had been her first acquaintances in Baron Street. With the aid of Mervyn, whose people hailed from the same county—work and a decent home were found for the pair, and a new chance was thus given them. This necessitated a vast amount of scribbling between the young lady and her ally before it was finally brought to a triumphant conclusion. Then there were the occupants of the beds in St. Placida's, for whose admission she had provided; there was that seamstress for whom she had found work, that kept her at least in bread; there were her friends the doll and cabinet makers; on the whole, there was matter enough to write about, and much too, to talk over when they met.

Mervyn was seldom persuaded to stay: to save or keep David Jardine even a little longer in life was beyond his skill, and he had too deep an interest in his work to neglect it, even for love's sake. But one perfect autumn day he yielded to temptation, and took Alicia out on the river. The glory of September was on the world, and he abandoned himself recklessly to enjoyment. But Alicia's eyes were sombre.

"It is beautiful; but I don't think it is quite right to enjoy it."

"It is right," he said, smiling at her morbidness. "I mean to shake the dust of the East off me, and enjoy it to the full. All work and no play makes Felix a dull boy. I don't know anything about Bethnal Green; I was never there in my life—never even heard of it. I am a happy tourist—till sunset."

"You have worked for it, and you deserve it."

"So have you."

"He'll live till his book has run the gauntlet of criticism," said Mervyn; "a good review might keep him in the world a day or two longer, but I won't answer for the effects of a bad one."

"Then there shall be no bad ones."

Mervyn smiled. "I'm afraid you can't compass that," he said. "The book is yours now, but you surrender your power over it when you give it to the world."

"At least, they can be kept from David," said Alicia.

"Oh, no; I have been playing for twenty-five years. I think I am tired of it."

"Don't you like being here? It seems to me such a jolly little box."

"No; I believe I hate it. It is a 'bijou residence'—the description is enough to condemn it. I don't like half-measures. I like being very splendid, or else"——

"Or else?" he questioned, with a new eagerness in his voice.

But Alicia was dipping her fingers in the water, and was silent on that business.

"A conscience is a very inconvenient possession," she said.

"If mine had slumbered peacefully after the fashion of a well-conducted conscience, I might have been wearing my fourth costume for the day at this moment and criticising the band and my neighbours' gowns at Baden-Baden. Think what a loss it is to have relinquished those joys, and to be no longer able to be wholly and completely frivolous and supercilious! Why was it ordained that I should have cousins—cousins who had the bad taste to drift to Bethnal Green and to draw me after them?"

"One of them will not trouble you long."

"And the other you would suggest I might abandon? Mary has done nothing—she is not even a poet. She is not even pretty. Why should I cleave to her?"

"There is always that alternative," said Mervyn, with a darkening brow. "You can leave us; you can go back again."

She looked at him calmly.

"Yes," she said, "there is that remedy. And it is quite possible I might learn to forget in time."

"If your conscience will permit you," said the young man gravely, but with laughter in his eye. And since he could laugh once more it is certain he was not very much afraid she would put her threat into execution.

So the weeks passed, and with the shortening autumn days, David Jardine grew so much weaker that it was clear, even to Mary, that he and the leaf must fall together. She prepared herself for that time, accepting sorrow and wearing it very gently.

David alone seemed to have no foreboding of coming change; his talk was all of what he and she were to do together.

"We shan't go back to Baron Street nor yet to Mrs. Carter's," he said. "You mind, Mary, I told you that first night it was only for a time, for a little while, till I should become known. We must move to a better quarter now, where we need not be ashamed to receive other visitors. I have been talking to Alicia, and she tells me that a great many authors and artists live in Kensington. We shall go there. I walked there once—it is a green and pretty place, almost with a look of the country. You will like that, Mary."

"Yes, dear, yes," said Mary, forcing back her tears.

"Sixty pounds isn't much, but it will do to start with, and I'll make a great deal more by my next book. Alicia tells me a first book never fetches much. She went for me, you know, to Short and Long to explain my wishes about the binding, and she says they told her that. They said, too, that poetry is never so well paid as prose. Well, I can write prose too. Never fear, Mary, my lass, I shall do bravely, and you shall live like a princess. You've been a good girl, Mary"——

"Oh, David, David," cried Mary, whose heart was breaking.

"Yes," he went on, encouraging her. "You've stuck by me and not complained, though you didn't like Baron Street. I could see that well enough. But you will like Kensington. And if you care to go and see Alicia and her father and sister when they come home, I don't mind now. They won't be ashamed of us any longer."

"Alicia never was ashamed of us," said Mary, gravely.

"No, that's true. She has a good deal of insight—for a girl. She prophesied from the first that I should succeed, and you see she was right. But the other one—the little one they call Kitty—she looked at us as if we were wild beasts. She never came back."

"Poor child," said Mary, gently. "It frightened her to come to such a place. It frightened me too, at first, David, till I came to know the people."

"Yes, I know," he smiled; "well, that's all over now, Mary. How astonished they'll be at home," he said after a pause. "I should like to see the schoolmaster's face when he hears of my book."

"Should you like to see him again, David?" she asked eagerly. "It would be like a bit of home."

"We'll go home some day," he said, "and see them all again. Next summer perhaps, when I have written another book."

This was how David spoke, and every word he said was a sword-stab to her loving heart. Sometimes she clutched wildly at a hope that he might be right and the others of them all wrong. Doctors were often mistaken—so she had heard. David might be going to live and to do great things after all. But one look at his face generally dispelled this dream. There was no mistaking its record.

The end came sooner, however, than either of them anticipated. It was on a day marked by two events, or rather, two arrivals, both of them exciting to the quiet little household.

The second comer was the schoolmaster from the North. Mary had kept up a diligent correspondence with this faithful friend, and he came now in answer to her summons.

Alicia looked at this rugged, composed son of the hills, with much interest; they had mutually heard of one another, and the nod he gave her on introduction, betokened a friendliness she was quite willing to reciprocate, while she recognised the independence it claimed.

She was not a fine lady to him, whatever she might be to others. She was a young lass who had been good to Mary, and it was on that ground he accepted the hand she offered.

Even to Mary his greeting was not effusive, but her stirred feelings moved tremulously in her face.

"How's the lad?" he asked.

"He is very happy," she said. "He will be glad to see you to-day, of all days."

He shook his head doubtfully. "I misdoubt it," he said. "If the lad could have made up his mind he was nae genius he might have been one by now. He'll not thole to hear that any better than before."

"But he is one now," said Mary, with a soft light in her eyes. "Dear master, you mustn't doubt him any longer—you won't when you hear. I did not tell you, for I meant it to be a great surprise, only, only—when David fell ill" (she hung her head) "I felt that I could not wait."

"What's the surprise, lass?"

"David has succeeded," she said, and she lifted her head once more proudly. "They have printed his book and given him money for it—a great deal of money. It came a little while ago—the first copy of his own book. I left him alone with it. You will go up and see him soon? He will be proud to show it to you."

Alicia had before this left them alone, divining that they must have much to say to each other. She wandered out upon the lawn; she could hear Mary's soft voice murmuring on, crossed now and then by a grunt of assent from the listener. This rugged person interested her, and she embarked in various speculations concerning him. It was one of the days when Mervyn was expected at the cottage, and she had a certain humorous pleasure in forecasting the meeting between doctor and dominie. She imagined they would "hit it off," as Mervyn would have phrased it, sharing as they did a common sincerity and singleness of purpose. When she thought of the schoolmaster and his former pupil—the poor poet, lying on his sick bed, engrossed in his own production—she found it less easy to fit them with a mutual standpoint.

"But then, David never liked him," she mused. "I suppose his bluntness hurt the boy's sensitive vanity. What a sad thing it is to be a poet! It must have been for Mary's sake he came all this long way—into the land of the enemy."

By-and-by she heard them go together to David's room, which he never quitted now. Mary went first, to prepare him for a visitor—for a "bit of home," as she had said.

It was a bright little chamber, clean and sweet and very still. The window was open and the sun looked in.

"David," she said softly, "here is an old friend come to see you."

The boy lay facing the sunlight, but he did not move or answer her. It was so still that you could almost hear the wash of the river beyond the little lawn.

"He is asleep," said Mary; but she looked at the schoolmaster with fear and a dawning dread in her eyes.

She took a step nearer.

"David! David!" she called once more, the dread growing in her eyes, but still he never stirred.

Then with a great wail of anguish she fell down on her knees beside him.

David was asleep with his cheek pillowed on the book—a smile on his lips—too fast asleep to hear the words of love or sympathy or praise that he had counted so sweet. Perhaps the joy of fulfilment coming after long despair had been too great for him to bear, and so had snapped the slender thread of his life.

Happy poet to die while his cup of delight was full—before discouragement, neglect, censure, had turned its sweetness into gall.

(*To be continued.*)

